# Synthesis and Cytotoxic Activity Evaluation of Novel Arylpiperazine Derivatives on Human Prostate Cancer Cell Lines

**DOI:** 10.3390/molecules190812048

**Published:** 2014-08-12

**Authors:** Hong Chen, Xue Liang, Fang Xu, Bingbing Xu, Xuelan He, Biyun Huang, Mu Yuan

**Affiliations:** Pharmaceutical Research Center, Guangzhou Medical University, 195# Dongfengxi Road, Guangzhou 510182, China; E-Mails: chenwexpo@sina.com (H.C.); liangliang4983@163.com (X.L.); sherrywon03@163.com (F.X.); yjxubb@163.com (B.X.); gzmc_xlhe@hotmail.com (X.H.); guangyihby@126.com (B.H.)

**Keywords:** synthesis, arylpiperazine derivatives, cytotoxic activity, CCK-8, structure-activity relationship

## Abstract

A series of novel arylpiperazine derivatives was synthesized. The *in vitro* cytotoxic activities of all synthesized compounds against three human prostate cancer cell lines (PC-3, LNCaP, and DU145) were evaluated by a CCK-8 assay. Compounds **9** and **15** exhibited strong cytotoxic activities against LNCaP cells (IC_50_ < 5 μM), and compound **8** (IC_50_ = 8.25 μM) possessed the most potent activity against DU145 cells. However, these compounds also exhibited cytotoxicity towards human epithelial prostate normal cells RWPE-1. The structure–activity relationship (SAR) of these arylpiperazine derivatives was also discussed based on the obtained experimental data.

## 1. Introduction

Prostate cancer is the most common non-skin cancer in men and is the second-leading cause of cancer-related deaths in the US [1]. The incidence of prostate cancer varies worldwide. Generally, the incidence rate in Occidental countries is higher than that in Asian countries [2,3]. Old age [4], ethnicity [4], family history [5,6] diet [7] hormones and other related factors [3] of this disease are the principal risk factors of developing prostate cancer. Prostate cancer mortality typically results from the metastasis to the bone and lymph nodes, as well as the progression from androgen-dependent to androgen-independent prostatic growth [8]. Clinically, localized disease is potentially curable [9] through surgery or radiotherapy to remove or destroy cancerous cells. However, metastatic prostate cancer remains essentially incurable and androgen ablation therapy has been the standard therapy [10,11]. Although various chemotherapeutic agents [12] are used solely or in combination with radiotherapy to treat advanced diseases, none of the conventional approaches to cancer therapy have been proven to be highly successful for prostate cancer. Other studies have shown once tumor cells have become hormone refractory, the standard cytotoxic agents for hormone-refractory prostate cancer (HRPC) can do little to improve the treatment outcomes or survival rates [13,14,15], although they can still relieve the pain to some extent in some patients. Therefore, inventing and developing more effective and safe anti-prostate cancer drugs are urgently needed.

**Figure 1:**
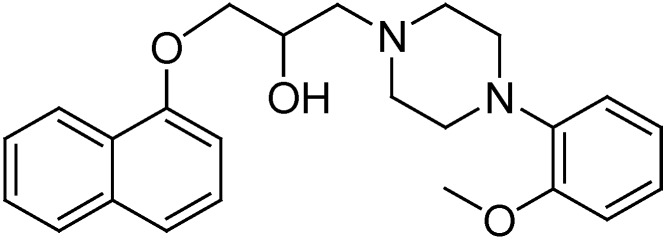
Structure of naftopidil.

Compounds with arylpiperazine moieties have a wide range of bioactivities including antiarrhythmic [16] diuretic [17], antiallergic [18], antidepressant [19] anxiolytic [20] antipsychotic [21] antimalarial [22] and antiplasmodial properties [23]. In addition, these compounds also exhibit receptor-blocking properties [17,24,25,26,27]. Naftopidil (Figure 1), an arylpiperazine compound, is a speciﬁc α1d receptor antagonist [28,29]. Previous studies have demonstrated that naftopidil has higher afﬁnity for the α1d-adrenergic receptor than for the α1a- and α1b-adrenergic receptor subtypes [30], and in Japan it is one of the most widely used α1-adrenergic receptor antagonists for the treatment of benign prostatic hyperplasia (BPH) [31,32]. 

**Scheme 1:**
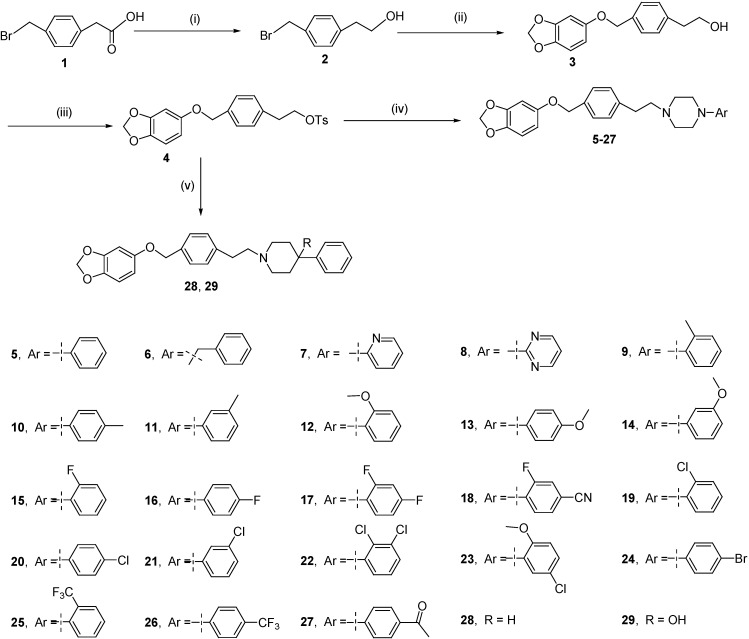
The synthesis route of compounds **5**–**29**.

Recent studies have shown that naftopidil could possibly exert an anticancer effect and inhibit prostate cancer cell growth by arresting the G1 cell cycle phase [33,34]. In addition, naftopidil can also decrease cell viability for bladder, prostate, and renal cancer cell lines [35], as well as induce apoptosis in malignant mesothelioma cell lines [36]. These findings indicate that naftopidil might be useful as an anti-cancer drug. Many studies have shown that arylpiperazine derivatives may act as potential α1a- and/or α1a- + α1d-selective ligands for the treatment of BPH. However, there have been few studies on cytotoxic activities of these compounds against human prostate cancer cells [37,38,39,40,41,42]. As part of our group’s continuing efforts to study the core framework of naftopidil [43,44,45,46], herein we report the synthesis of a series of novel arylpiperazinyl derivatives based on naftopidil (Scheme 1) to identify new anti-prostate cancer drug candidates with potential for further development. All synthesized compounds were evaluated for their cytotoxic activities against the androgen-insensitive human prostate cancer cell line PC-3, the androgen-sensitive human prostate cancer cell line LNCaP, the androgen-insensitive human prostate cancer cell line DU145, and the human prostate epithelial cell line RWPE-1. The SAR was further discussed on the basis of the obtained experimental data. Some compounds exhibited strong anti-cancer activities against the tested cancer cells and superior potency than naftopidil.

*Reagents and conditions*: (i) BH_3_.S(CH_3_)_2_, THF, 0 °C for 1 h, and room temperature for 10 h; (ii) sesamol, K_2_CO_3_, CH_3_CN, reflux, 16 h; (iii) TsCl, Et_3_N and 4-dimethylaminopyridine, Cl_2_CH_2_, 0 °C, 16 h; (iv) arylpiperazines, K_2_CO_3_, CH_3_CN, reflux, 16 h; (v) phenylpiperidines, K_2_CO_3_, CH_3_CN, reflux, 16 h.

## 2. Results and Discussion

### 2.1. Chemistry

As depicted in Scheme 1, a series of novel arylpiperazine derivatives were synthesized in four steps starting from the commercially available 2-(4-(bromomethyl)phenyl)acetic acid (**1**). First, compound **1** was reduced to alcohol **2** in the presence of a borane–methyl sulfide complex (2 M in tetrahydrofuran) at 0 °C for 1 h and then at room temperature for 10 h. The intermediate **2** was directly used without further purification. The nucleophilic substitution reaction of compound **2** with sesamol in the presence of potassium carbonate (K_2_CO_3_) gave compound **3** (67% yield from compound **1**) after 16 h at reflux. Compound **3** was treated with 4-toluenesulfonyl chloride in the presence of triethylamine and a catalytic amount of 4-dimethylaminopyridine at 0 °C for 16 h to generate compound **4** (95% yield). Finally, the reactions of compound **4** with various arylpiperazines or phenyl-piperidines in the presence of K_2_CO_3_ at reflux for 16 h gave arylpiperazine or phenylpiperidine derivatives **5** to **29** (55% to 82% yield; Scheme 1). The structures of all the compounds (as their HCl salts) were confirmed using ^1^H-NMR, ^13^C-NMR, and elemental analyses (C, H, and N).

### 2.2. Cytotoxic Activity

The synthesized target compounds **5** to **29** were evaluated for their *in vitro* cytotoxic activities against the three human prostate cancer cell lines (PC-3, LNCaP, and DU145), and compared with their effects on human prostate epithelial cell line RWPE-1 by CCK-8 assay [47,48,49]. The results are summarized in Table 1.

**Table 1:** *In vitro* cytotoxicity of compounds **5**–**29**.

Compd.	IC_50_ (μM) ^a^			
	PC-3 ^b^	LNCaP ^b^	DU145 ^b^	RWPE-1 ^b^
**5**	>50	27.85 ± 1.72	>50	>50
**6**	>50	27.70 ± 4.17	28.92 ± 0.98	38.78 ± 0.77
**7**	>50	31.22 ± 0.63	>50	41.05 ± 0.54
**8**	>50	26.60 ± 0.86	8.25 ± 0.16	31.69 ± 3.14
**9**	>50	3.47 ± 0.36	>50	31.01 ± 1.57
**10**	>50	31.94 ± 0.89	47.75 ± 2.02	>50
**11**	>50	>50	>50	>50
**12**	>50	30.73 ± 0.83	>50	>50
**13**	>50	28.27 ± 0.49	>50	>50
**14**	>50	>50	>50	38.95 ± 0.77
**15**	>50	1.25 ± 0.23	>50	41.01 ± 0.47
**16**	>50	32.47 ± 1.04	>50	>50
**17**	>50	26.76 ± 1.00	26.83 ± 0.92	23.94 ± 0.75
**18**	26.89 ± 1.00	13.11 ± 0.51	>50	>50
**19**	>50	26.58 ± 0.40	>50	29.85 ± 0.80
**20**	>50	>50	>50	>50
**21**	>50	25.28 ± 1.55	>50	>50
**22**	>50	39.41 ± 0.54	>50	>50
**23**	>50	33.25 ± 2.69	>50	35.89 ± 1.25
**24**	>50	19.85 ± 1.01	>50	>50
**25**	>50	32.96 ± 0.21	>50	18.94 ± 3.68
**26**	>50	26.83 ± 1.51	>50	>50
**27**	24.95 ± 0.68	>50	>50	26.79 ± 3.01
**28**	>50	18.21 ± 1.88	24.71 ± 2.22	28.46 ± 2.18
**29**	>50	42.98 ± 7.58	38.23 ± 2.45	19.73 ± 0.01
naftopidil	42.10 ± 0.79	22.36 ± 0.61	34.58 ± 0.31	>50

^a^ IC_50_ values are taken as means ± standard deviation from three experiments; ^b^ PC-3, LNCaP and DU145, human prostate cancer cell line; RWPE-1, the human prostate epithelial cell line.

As shown in Table 1, some compounds exhibited moderate to strong cytotoxic activities against the tested cancer cell lines, and even exhibited much better activity than naftopidil. For example, compounds **9** and **15** exhibited strong cytotoxic activities against LNCaP cells (IC_50_ < 5 μM) and these compounds showed excellent selective activity for LNCaP cells over the other tested cancer cells. However, compounds **9** and **15** exhibited cytotoxic effects on normal RWPE-1 human epithelial prostate cells. Moreover, compound **8** (IC_50_ = 8.25 μM) showed the most potent activity against DU145 cells and a marked selectivity for DU145 cells over the other tested cancer cells.

The SAR analysis revealed the following: (1) phenylpiperazine derivative **5** exhibited similar cytotoxic activities against LNCaP cells compared with benzylpiperazine derivative **6**, however, these activities decreased significantly in DU145 cells; (2) compound **8** exhibited a more effective cytotoxic activity than compounds **5** and **7** against DU145 cells. These results suggest that the introduction of a pyrimidinyl moiety at the 4-position of piperazine ring was beneficial for anti-cancer activity. Moreover, compound **8** showed excellent selective activity for DU145 cells over the other tested cancer cells; (3) compound **11** lost potency (IC_50_ > 50 μM) against LNCaP cells compared with compounds **9** and **10** (IC_50_ = 3.47 and 31.94 μM, respectively). The activity profiles indicated that a methyl at the *m*-position on the phenyl group was inauspicious for anti-cancer activity. A similar potency profile was observed in compounds **12**, **13**, and **14**; (4) Moreover, compounds containing an *o*-methyl-substituted phenyl group showed better activity for LNCaP cells than did the *p*-methyl-substituted group for LNCaP cells, as exemplified by compound **9** which showed significantly improved activity, while compound **10** exhibited moderate cytotoxic activity. A similar potency profile was also observed in compound **15** (IC_50_ = 1.25 μM) *vs.* **16** (IC_50_ = 32.47 μM), as well as **19** (IC_50_ = 26.58 μM) *vs.* **20** (IC_50_ > 50 μM); (5) Compared to compounds **16** and **24** (IC_50_ = 19.85 μM), compound **20** (IC_50_ > 50 μM) lost potency against LNCaP cells. The obtained results suggested that a chloro group in the *p*-position on the phenyl group was not favorable for anti-cancer activity; (6) Although compounds **22** (IC_50_ = 39.41 μM), **23** (IC_50_ = 33.25 μM), **25** (IC_50_ = 32.96 μM), and **26** (IC_50_ = 26.83 μM) demonstrated moderate cytotoxic activities against LNCaP cells, these compounds had improved selectivity for LNCaP cells over the other cancer cells; (7) The compounds with methyl or fluoro groups at the *o*-position on the phenyl group exhibited a relatively strong cytotoxicity against LNCaP cells (IC_50_ < 5 μM); (8) Phenylpiperidine derivatives **28** and **29** (Scheme 1) were synthesized to compare the anti-cancer activity of arylpiperazine derivatives. As shown in Table 1, compounds **28** and **29** exhibited moderate cytotoxic activities against LNCaP and DU145 cells. However, these compounds also exhibited cytotoxic effect on human prostate epithelial cell line. 

## 3. Experimental Section

### 3.1. General Information

Reagents and solvents were commercially available. Solvents were dried and purified prior to use using standard procedures. Melting points were determined on a SGW X-4 micro melting point apparatus (Shanghai Precision & Scientific Instrument Co., Ltd, Shanghai, China) and are uncorrected. NMR spectra were determined on a Bruker AV-400 NB spectrometer (Faellanden, Switzerland) in DMSO-*d*_6_ using TMS as internal standard, and coupling constants (*J*) are in Hz. EI mass spectra were recorded on a DSQ mass spectrometer. Elemental analyses (C, H, N) were performed on an Elementar Vario EL elemental analyzer and the analytical results were within ±0.4% of the theoretical values for the formula given unless otherwise listed. Flash column chromatography was performed with silica gel (300–400 mesh, Qing Dao Ocean Chemical Factory, Qingdao, China) eluted with petroleum ether–ethyl acetate.

#### 3.1.1. 2-(4-(Bromomethyl)phenyl)ethanol (**2**)

To a cooled (0 °C) solution of carboxylic acid **1** (5 g, 0.021 mol) in dry tetrahydrofuran (THF, 100 mL) borane-dimethyl sulfide complex (21.9 mL, 0.042 mol, 2 M in THF) was added dropwise. The reaction mixture was stirred at 0 °C for 1 h and at room temperature for 10 h. Water (20 mL) was added slowly and extracted with ethyl acetate (3 × 100 mL). The combined organic phase was successively washed with water, brine, dried over anhydrous magnesium sulfate, and concentrated *in vacuo*. The resulting residue was directly used without further purification in the following step.

#### 3.1.2. 2-(4-((Benzo[d][1,3]dioxol-5-yloxy)methyl)phenyl)ethanol (**3**)

To a solution of compound **2** (4 g, 18.7 mmol) in acetone (100 mL) sesamol (2.58 g, 18.7 mmol）and potassium carbonate (10.32 g, 74.8 mmol) were added, and the reaction mixture was stirred at reflux for 16 h. After cooling to ambient temperature, the reaction mixture was filtered through a Buchner funnel. After filtration the filtrate was concentrated *in vacuo* and the residue was purified by silica gel column chromatography using ethyl acetate/petroleum ether (1/10, *v*/*v*) as eluent to afford 4.06 g of compound **3** (67% from compound **1**) as a white solid. m.p. 102–103 °C; ^1^H-NMR (400 MHz, DMSO-*d*_6_) δ in ppm: 7.32 (d, *J* = 8.0 Hz, 2H, Ar-H), 7.22 (d, *J *= 8.0 Hz, 2H, Ar-H), 6.79 (d, *J *= 8.5 Hz, 1H, Ar-H), 6.68 (d, J = 2.5 Hz, 1H, Ar-H), 6.43 (dd, J = 8.5, 2.5 Hz, 1H, Ar-H), 5.94 (s, 2H, CH_2_), 4.97 (s, 2H, CH_2_), 4.60 (t, *J *= 5.2 Hz, 1H, OH), 3.60 (td, *J* = 7.0, 5.2 Hz, 2H, CH_2_), 2.72 (t, *J* = 7.0 Hz, 2H, CH_2_); ^13^C-NMR (101 MHz, DMSO-*d*_6_) δ in ppm: 154.25, 148.34, 141.64, 139.65, 135.07, 129.33, 128.09, 108.46, 106.64, 101.42, 98.55, 70.39, 62.57; MS (EI, *m/z*): 272 (M^+^), 167, 149, 135 (100%), 117, 105, 97, 79.

#### 3.1.3. 2-(4-((Benzo[d][1,3]dioxol-5-yloxy)methyl)phenyl)ethyl 4-methylbenzenesulfonate (**4**)

To a solution of compound **3** (4 g, 14.7 mmol), triethylamine (5.94 g, 58.8 mmol) and 4-dimethyl- aminopyridine (0.18 g, 1.47 mmol) in dry dichloromethane (CH_2_Cl_2_, 100 mL) at 0 °C was added dropwise a solution of 4-toluenesulfonyl chloride (4.19 g, 22.1 mmol) in CH_2_Cl_2_ (10 mL). The reaction mixture was stirred at 0 °C for 16 h. Water (20 mL) was added slowly and the reaction mixture was extracted with CH_2_Cl_2_ (3 × 100 mL). The combined organic phase was successively washed with water and brine, dried over anhydrous magnesium sulfate, and concentrated *in vacuo*. The residue was purified by silica gel column chromatography using ethyl acetate/petroleum ether (1/15, *v*/*v*) as eluent to afford 5.76 g (95%) of compound **4** as a white solid. m.p. 90–91 °C; ^1^H-NMR (400 MHz, DMSO-*d*_6_) δ in ppm: 7.65 (d, *J* = 8.0 Hz, 2H, Ar-H), 7.40 (d, *J* = 8.0 Hz, 2H, Ar-H), 7.30 (d, *J* = 8.0 Hz, 2H, Ar-H), 7.15 (d, *J* = 8.0 Hz, 2H, Ar-H), 6.80 (d, *J* = 8.5 Hz, 1H, Ar-H), 6.69 (d, *J* = 2.5 Hz, 1H, Ar-H), 6.44 (dd, *J *= 8.5, 2.5 Hz, 1H, Ar-H), 5.95 (s, 2H, CH_2_), 4.98 (s, 2H, CH_2_), 4.23 (t, *J* = 6.4 Hz, 2H, CH_2_), 2.89 (t, *J* = 6.4 Hz, 2H, CH_2_), 2.40 (s, 3H, CH_3_); ^13^C-NMR (101 MHz, DMSO-*d*_6_) δ in ppm: 154.22, 148.36, 145.23, 141.68, 136.78, 135.90, 132.78, 130.52, 129.28, 128.21, 127.89, 108.47, 106.64, 101.44, 98.55, 71.43, 70.27, 34.55, 21.53; MS (EI, *m/z*): 426 (M^+^), 289, 254, 155, 137, 117 (100%), 104, 91.

#### 3.1.4. General Procedure for the Preparation of Compounds **5**–**29**

To a solution of **4** (100 mg, 0.23 mmol) in acetonitrile (CH_3_CN, 10 mL) was added the corresponding arylpiperazine or phenylpiperidine (1.2 equiv) and potassium carbonate (6.0 equiv). The reaction mixture was stirred at reflux for 16 h. After cooling to ambient temperature, the reaction mixture was filtered through a Buchner funnel. After filtration the filtrate was concentrated *in vacuo* and the residue was purified by silica gel column chromatography using ethyl acetate/petroleum ether (1/5, *v*/*v*) as eluent to afford the corresponding products, and all compounds were recrystallized from trichloromethane and *n*-hexane.

*1-(2-(4-((Benzo[d][1,3]dioxol-5-yloxy)methyl)phenyl)ethyl)-4-phenylpiperazine* (**5**)*.* Yield: 65%, m.p. 195–196 °C (HCl salt); ^1^H-NMR (400 MHz, DMSO-*d*_6_) δ in ppm: 11.51 (s, 1H, N^+^H), 7.40 (d, *J* = 8.0 Hz, 2H, Ar-H), 7.30 (d, *J* = 8.0 Hz, 2H, Ar-H), 7.27 (dd, *J* = 8.0, 7.3 Hz, 2H, Ar-H), 7.02 (d, *J* = 8.0 Hz, 2H, Ar-H), 6.87 (t, *J* = 7.3 Hz, 1H, Ar-H ), 6.80 (d, *J* = 8.5 Hz, 1H, Ar-H), 6.68 (d, *J* = 2.5 Hz, 1H, Ar-H), 6.43 (dd, *J* = 8.5, 2.5 Hz, 1H, Ar-H), 5.95 (s, 2H, CH_2_), 5.00 (s, 2H, CH_2_), 3.82 (d, *J* = 10.4 Hz, 2H, CH_2_), 3.62 (d, *J* = 10.4 Hz, 2H, CH_2_), 3.40–3.09 (m, 8H, CH_2_); ^13^C-NMR (101 MHz, DMSO-*d*_6_) δ in ppm: 154.07, 149.87, 148.26, 141.60, 137.07, 136.08, 129.49, 129.09, 128.40, 120.45, 116.40, 108.38, 106.61, 101.36, 98.49, 70.13, 56.36, 50.92, 45.80, 29.33; Anal. Calc. for C_26_H_28_N_2_O_3_·2HCl: C, 63.80; H, 6.18; N, 5.72. Found: C, 63.64; H, 6.12; N, 5.58.

*1-(2-(4-((Benzo[d][1,3]dioxol-5-yloxy)methyl)phenyl)ethyl)-4-benzylpiperazine* (**6**)*.* Yield: 80%, m.p. 177–178 °C (HCl salt); ^1^H-NMR (400 MHz, DMSO-*d*_6_) δ in ppm: 12.00 (s, 1H, N^+^H), 7.71–7.42 (m, 5H, Ar-H), 7.39 (d, *J* = 8.0 Hz, 2H, Ar-H), 7.30 (d, *J* = 8.0 Hz, 2H, Ar-H), 6.80 (d, *J* = 8.5 Hz, 1H, Ar-H), 6.68 (d, *J* = 2.5 Hz, 1H, Ar-H), 6.43 (dd, *J* = 8.5, 2.5 Hz, 1H, Ar-H), 5.95 (s, 2H, CH_2_), 5.00 (s, 2H, CH_2_), 4.34 (s, 2H, CH_2_), 3.94–2.93 (m, 12H, CH_2_); Anal. Calc. for C_27_H_30_N_2_O_3_·2HCl: C, 64.41; H, 6.41; N, 5.56. Found: C, 63.98; H, 6.45; N, 5.31.

*1-(2-(4-((Benzo[d][1,3]dioxol-5-yloxy)methyl)phenyl)ethyl)-4-(pyridin-2-yl)piperazine* (**7**). Yield: 70%, m.p. 185–186 °C (HCl salt); ^1^H-NMR (400 MHz, DMSO-*d*_6_) δ in ppm: 11.92 (s, 1H, N^+^H), 8.13 (dd, *J* = 5.6, 1.2 Hz, 1H, pyridine H), 7.96 (t, *J* = 7.6 Hz, 1H, pyridine H), 7.41–7.30 (m, 5H, Ar-H and pyridine H ), 6.98 (t, *J* = 6.4 Hz, 1H, pyridine H), 6.80 (d, *J* = 8.5 Hz, 1H, Ar-H), 6.69 (d, *J* = 2.5 Hz, 1H, Ar-H), 6.44 (dd, *J* = 8.5, 2.5 Hz, 1H, Ar-H), 5.95 (s, 2H, CH_2_), 5.00 (s, 2H, CH_2_), 4.54 (d, *J* = 10.6 Hz, 2H, CH_2_), 3.78–3.09 (m, 10H, CH_2_); ^13^C-NMR (101 MHz, DMSO-*d*_6_) δ in ppm: 154.16, 148.35, 141.69, 137.09, 136.19, 129.17, 128.50, 114.40, 108.47, 106.69, 101.45, 98.58, 70.22, 56.47, 50.38, 43.40, 40.66, 29.38; Anal. Calc. for C_25_H_27_N_3_O_3_·2.8HCl: C, 57.79; H, 5.78; N, 8.09. Found: C, 57.78; H, 6.06; N, 7.86.

*2-(4-(2-(4-((Benzo[d][1,3]dioxol-5-yloxy)methyl)phenyl)ethyl)piperazin-1-yl)pyrimidine* (**8**). Yield: 65%, m.p. 181–182 °C (HCl salt); ^1^H-NMR (400 MHz, DMSO-*d*_6_) δ in ppm: 11.36 (s, 1H, N^+^H), 8.45 (d, *J* = 4.4 Hz, 2H, pyrimidine H), 7.39 (d, *J* = 7.5 Hz, 2H, Ar-H), 7.29 (d, *J* = 7.5 Hz, 2H, Ar-H), 6.85–6.37 (m, 4H, Ar-H and pyrimidine H), 5.94 (s, 2H, CH_2_), 5.00 (s, 2H, CH_2_), 4.70 (d, *J* = 11.4 Hz, 2H, CH_2_), 3.64–3.05 (m, 10H, CH_2_); ^13^C-NMR (101 MHz, DMSO-*d*_6_) δ in ppm: 161.07, 158.61, 154.14, 145.81, 141.68, 137.02, 136.20, 129.16, 128.49, 111.79, 108.46, 106.69, 101.45, 98.57, 70.20, 56.59, 50.85, 29.41; Anal. Calc. for C_24_H_26_N_4_O_3_·1.5HCl: C, 60.92; H, 5.86; N, 11.84. Found: C, 60.67; H, 5.98; N, 11.61.

*1-(2-(4-((Benzo[d][1,3]dioxol-5-yloxy)methyl)phenyl)ethyl)-4-o-tolylpiperazine* (**9**)*.* Yield: 80%, m.p. 202–203 °C (HCl salt); ^1^H-NMR (400 MHz, DMSO-*d*_6_) δ in ppm: 11.16 (s, 1H, N^+^H), 7.42 (d, *J* = 8.1 Hz, 2H, Ar-H), 7.32 (d, *J* = 8.1 Hz, 2H, Ar-H), 7.10 (m, 4H, Ar-H), 6.81 (d, *J* = 8.5 Hz, 1H, Ar-H), 6.69 (d, *J* = 2.5 Hz, 1H, Ar-H), 6.44 (dd, *J* = 8.5, 2.5 Hz, 1H, Ar-H), 5.96 (s, 2H, CH_2_), 5.01 (s, 2H, CH_2_), 3.62 (d, *J* = 10.9 Hz, 2H, CH_2_), 3.43–3.08 (m, 10H, CH_2_), 2.28 (s, 3H, CH_3_); ^13^C-NMR (101 MHz, DMSO-*d*_6_) δ in ppm: 155.55, 151.59, 149.74, 143.08, 138.51, 137.58, 133.84, 132.86, 130.58, 129.89, 128.55, 125.62, 120.84, 109.85, 108.08, 102.83, 99.96, 71.59, 57.99, 53.31, 50.02, 30.88, 19.26; Anal. Calc. for C_27_H_30_N_2_O_3_·2HCl: C, 64.41; H, 6.41; N, 5.56. Found: 64.63; H, 6.45; N, 5.44.

*1-(2-(4-((Benzo[d][1,3]dioxol-5-yloxy)methyl)phenyl)ethyl)-4-p-tolylpiperazine* (**10**). Yield: 70%, m.p. 184–185 °C (HCl salt); ^1^H-NMR (400 MHz, DMSO-*d*_6_) δ in ppm: 11.30 (s, 1H, N^+^H), 7.40 (d, *J* = 8.0 Hz, 2H, Ar-H), 7.30 (d, *J* = 8.0 Hz, 2H, Ar-H), 7.08 (d, *J* = 8.3 Hz, 2H, Ar-H), 6.92 (d, *J* = 8.3 Hz, 2H, Ar-H), 6.79 (d, *J* = 8.5 Hz, 1H, Ar-H), 6.68 (d, *J* = 2.5 Hz, 1H, Ar-H), 6.43 (dd, *J* = 8.5, 2.5 Hz, 1H, Ar-H), 5.94 (s, 2H, CH_2_), 5.00 (s, 2H, CH_2_), 3.75 (d, *J* = 9.2 Hz, 2H, CH_2_), 3.62 (d, *J* = 9.2 Hz, 2H, CH_2_), 3.40–3.07 (m, 8H, CH_2_), 2.22 (s, 3H, CH_3_); ^13^C-NMR (101 MHz, DMSO-*d*_6_) δ in ppm: 154.15, 148.34, 147.73, 141.69, 137.12, 136.17, 130.02, 129.62, 129.18, 128.49, 116.79, 108.47, 106.71, 101.45, 98.58, 70.22, 56.48, 51.05, 46.41, 29.44, 20.51; Anal. Calc. for C_27_H_30_N_2_O_3_·2HCl: C, 64.41; H, 6.41; N, 5.56. Found: C, 64.40; H, 6.43; N, 5.36.

*1-(2-(4-((Benzo[d][1,3]dioxol-5-yloxy)methyl)phenyl)ethyl)-4-m-tolylpiperazine* (**11**)*.* Yield: 82%, m.p. 171–172 °C (HCl salt); ^1^H-NMR (400 MHz, DMSO-*d*_6_) δ in ppm: 11.61 (s, 1H, N^+^H), 7.40 (d, *J* = 8.0 Hz, 2H, Ar-H), 7.30 (d, *J* = 8.0 Hz, 2H, Ar-H), 7.15 (t, *J* = 7.6 Hz, 1H, Ar-H), 6.93–6.62 (m, 5H, Ar-H), 6.43 (dd, *J* = 8.5, 2.5 Hz, 1H, Ar-H), 5.95 (s, 2H, CH_2_), 5.00 (s, 2H, CH_2_), 3.80 (d, *J* = 11.2 Hz, 2H, CH_2_), 3.62 (d, *J* = 11.2 Hz, 2H, CH_2_), 3.41–3.06 (m, 8H, CH_2_), 2.27 (s, 3H, CH_3_); ^13^C-NMR (101 MHz, DMSO-*d*_6_) δ in ppm: 154.17, 149.89, 148.35, 141.69, 138.78, 137.18, 136.15, 129.42, 129.17, 128.49, 121.45, 117.19, 113.80, 108.47, 106.70, 101.45, 98.58, 70.23, 56.45, 50.97, 46.01, 29.41, 21.80; Anal. Calc. for C_27_H_30_N_2_O_3_·2HCl: C, 64.41; H, 6.41; N, 5.56. Found: C, 64.54; H, 6.44; N, 5.44.

*1-(2-(4-((Benzo[d][1,3]dioxol-5-yloxy)methyl)phenyl)ethyl)-4-(2-methoxyphenyl)piperazine* (**12**). Yield: 75%, m.p. 173–174 °C (HCl salt); ^1^H-NMR (400 MHz, DMSO-*d*_6_) δ in ppm: 11.50 (s, 1H, N^+^H), 7.40 (d, *J* = 8.0 Hz, 2H, Ar-H), 7.31 (d, *J* = 8.0 Hz, 2H, Ar-H), 7.07–6.88 (m, 4H, Ar-H), 6.80 (d, *J* = 8.5 Hz, 1H, Ar-H), 6.69 (d, *J* = 2.5 Hz, 1H, Ar-H), 6.43 (dd, *J* = 8.5, 2.5 Hz, 1H, Ar-H), 5.95 (s, 2H, CH_2_), 5.01 (s, 2H, CH_2_), 3.80 (s, 3H, OCH_3_), 3.62 (d, *J* = 10.8 Hz, 2H, CH_2_), 3.51 (d, *J* = 10.8 Hz, 2H, CH_2_), 3.39–3.10 (m, 8H, CH_2_); ^13^C-NMR (101 MHz, DMSO-*d*_6_) δ in ppm: 154.16, 152.31, 148.35, 141.69, 139.69, 137.17, 136.17, 129.18, 128.49, 124.06, 121.33, 118.80, 112.50, 108.47, 106.70, 101.45, 98.58, 70.22, 56.62, 55.88, 51.52, 47.36, 29.43; Anal. Calc. for C_27_H_30_N_2_O_4_·2HCl: C, 62.43; H, 6.21; N, 5.39. Found: C, 62.37; H, 6.19; N, 5.25.

*1-(2-(4-((Benzo[d][1,3]dioxol-5-yloxy)methyl)phenyl)ethyl)-4-(4-methoxyphenyl)piperazine* (**13**)*.* Yield: 70%, m.p. 174–175 °C (HCl salt); ^1^H-NMR (400 MHz, DMSO-*d*_6_) δ in ppm: 11.62 (s, 1H, N^+^H), 7.40 (d, *J* = 8.0 Hz, 2H, Ar-H), 7.31 (d, *J* = 8.0 Hz, 2H, Ar-H), 7.05 (d, *J* = 9.0 Hz, 2H, Ar-H), 6.89 (d, *J* = 9.0 Hz, 2H, Ar-H), 6.80 (d, *J* = 8.5 Hz, 1H, Ar-H), 6.68 (d, *J* = 2.5 Hz, 1H, Ar-H), 6.43 (dd, *J* = 8.5, 2.5 Hz, 1H, Ar-H), 5.95 (s, 2H, CH_2_), 5.00 (s, 2H, CH_2_), 3.71 (s, 3H, OCH_3_), 3.66 (t, *J* = 9.0 Hz, 4H, CH_2_), 3.40–3.10 (m, 8H, CH_2_); ^13^C-NMR (101 MHz, DMSO-*d*_6_) δ in ppm: 154.30, 153.66, 147.84, 142.69, 141.19, 136.63, 135.66, 128.68, 127.98, 118.48, 114.47, 107.96, 106.19, 100.95, 98.08, 69.72, 55.88, 55.25, 50.41, 47.06, 28.93; Anal. Calc. for C_27_H_30_N_2_O_4_·1.9HCl: C, 62.87; H, 6.23; N, 5.43. Found: C, 62.90; H, 6.20; N, 5.25.

*1-(2-(4-((Benzo[d][1,3]dioxol-5-yloxy)methyl)phenyl)ethyl)-4-(3-methoxyphenyl)piperazine* (**14**)*.* Yield: 72%, m.p. 171–172 °C (HCl salt); ^1^H-NMR (400 MHz, DMSO-*d*_6_) δ in ppm: 11.56 (s, 1H, N^+^H), 7.40 (d, *J* = 8.0 Hz, 2H, Ar-H), 7.30 (d, *J* = 8.0 Hz, 2H, Ar-H), 7.17 (t, *J* = 8.0 Hz, 1H, Ar-H), 6.80 (d, *J* = 8.4 Hz, 1H, Ar-H), 6.69 (d, *J* = 2.4 Hz, 1H, Ar-H), 6.60 (dd, *J* = 8.4, 2.0 Hz, 1H, Ar-H), 6.55 (t, *J* = 2.0 Hz, 1H, Ar-H), 6.50–6.37 (m, 2H, Ar-H), 5.95 (s, 2H, CH_2_), 5.00 (s, 2H, CH_2_), 3.83 (d, *J* = 11.2 Hz, 2H, CH_2_), 3.74 (s, 3H, OCH_3_), 3.61 (d, *J* = 11.2 Hz, 2H, CH_2_), 3.41–3.06 (m, 8H, CH_2_); ^13^C-NMR (101 MHz, DMSO-*d*_6_) δ in ppm: 160.46, 153.86, 151.00, 148.05, 141.39, 136.88, 135.86, 130.03, 128.87, 128.19, 108.60, 108.17, 106.40, 105.53, 102.39, 101.15, 98.28, 69.93, 56.15, 55.18, 50.65, 45.51, 29.11; Anal. Calc. for C_27_H_30_N_2_O_4_·2HCl: C, 62.43; H, 6.21; N, 5.39. Found: C, 62.41; H, 6.19; N, 5.27.

*1-(2-(4-((Benzo[d][1,3]dioxol-5-yloxy)methyl)phenyl)ethyl)-4-(2-fluorophenyl)piperazine* (**15**)*.* Yield: 65%, m.p. 187–188 °C (HCl salt); ^1^H-NMR (400 MHz, DMSO-*d*_6_) δ in ppm: 11.46 (s, 1H, N^+^H), 7.40 (d, *J* = 8.0 Hz, 2H, Ar-H), 7.30 (d, *J* = 8.0 Hz, 2H, Ar-H), 7.23–6.96 (m, 4H, Ar-H), 6.79 (d, *J* = 8.5 Hz, 1H, Ar-H), 6.68 (d, *J* = 2.5 Hz, 1H, Ar-H), 6.43 (dd, *J* = 8.5, 2.5 Hz, 1H, Ar-H), 5.94 (s, 2H, CH_2_), 5.00 (s, 2H, CH_2_), 3.63 (d, *J* = 9.7 Hz, 2H, CH_2_), 3.50 (d, *J* = 9.7 Hz, 2H, CH_2_), 3.42–3.09 (m, 8H, CH_2_); ^13^C-NMR (101 MHz, DMSO-*d*_6_) δ in ppm: 156.27, 153.89, 148.08, 141.42, 138.50, 138.42, 136.83, 135.91, 128.91, 128.22, 125.18, 125.16, 123.65, 123.58, 119.80, 116.44, 116.24, 108.20, 106.44, 101.18, 98.31, 69.95, 56.31, 51.05, 47.16, 29.13; Anal. Calc. for C_26_H_27_FN_2_O_3_·2HCl: C, 61.54; H, 5.76; N, 5.52. Found: C, 61.38; H, 5.56; N, 5.33.

*1-(2-(4-((Benzo[d][1,3]dioxol-5-yloxy)methyl)phenyl)ethyl)-4-(4-fluorophenyl)piperazine* (**16**)*.* Yield: 60%, m.p. 196–197 °C (HCl salt); ^1^H-NMR (400 MHz, DMSO-*d*_6_) δ in ppm: 11.55 (s, 1H, N^+^H), 7.40 (d, *J* = 7.9 Hz, 2H, Ar-H), 7.30 (d, *J* = 7.9 Hz, 2H, Ar-H), 7.15–7.00 (m, 4H, Ar-H), 6.79 (d, *J* = 8.5 Hz, 1H, Ar-H), 6.68 (d, *J* = 2.5 Hz, 1H, Ar-H), 6.43 (dd, *J* = 8.5, 2.5 Hz, 1H, Ar-H), 5.94 (s, 2H, CH_2_), 5.00 (s, 2H, CH_2_), 3.74 (d, *J* = 8.8 Hz, 2H, CH_2_), 3.62 (d, *J* = 8.8 Hz, 2H, CH_2_), 3.40–3.08 (m, 8H, CH_2_); ^13^C-NMR (101 MHz, DMSO-*d*_6_) δ in ppm: 157.75, 155.40, 153.64, 147.83, 146.32, 141.17, 136.65, 135.64, 128.65, 127.97, 117.90, 117.82, 115.55, 115.33, 107.95, 106.18, 100.94, 98.06, 69.71, 55.90, 50.51, 46.07, 28.90; Anal. Calc. for C_26_H_27_FN_2_O_3_·1.9HCl: C, 61.99; H, 5.78; N, 5.56. Found: C, 62.02; H, 5.77; N, 5.41.

*1-(2-(4-((Benzo[d][1,3]dioxol-5-yloxy)methyl)phenyl)ethyl)-4-(2,4-difluorophenyl)piperazine* (**17**)*.* Yield: 67%, m.p. 169–170 °C (HCl salt); ^1^H-NMR (400 MHz, DMSO-*d*_6_) δ in ppm: 11.42 (s, 1H, N^+^H), 7.40 (d, *J* = 8.0 Hz, 2H, Ar-H), 7.30 (d, *J* = 8.0 Hz, 2H, Ar-H), 7.28–7.00 (m, 3H, Ar-H), 6.80 (d, *J* = 8.5 Hz, 1H, Ar-H), 6.68 (d, *J* = 2.5 Hz, 1H, Ar-H), 6.43 (dd, *J* = 8.5, 2.5 Hz, 1H, Ar-H), 5.95 (s, 2H, CH_2_), 5.00 (s, 2H, CH_2_), 3.63 (d, *J* = 8.6 Hz, 2H, CH_2_), 3.43 (d, *J* = 8.6 Hz, 2H, CH_2_), 3.40–3.09 (m, 8H, CH_2_); ^13^C-NMR (101 MHz, DMSO-*d*_6_) δ in ppm: 158.98, 158.93, 158.86, 156.58, 156.47, 156.24, 156.12, 153.84, 148.04, 141.38, 136.77, 135.88, 135.37, 135.34, 135.28, 135.25, 128.86, 128.18, 120.82, 120.80, 120.74, 120.70, 111.50, 111.47, 111.28, 111.26, 108.15, 106.38, 105.24, 104.98, 104.73, 101.14, 98.26, 69.90, 56.23, 51.03, 47.44, 29.10; Anal. Calc. for C_26_H_26_F_2_N_2_O_3_·1.25HCl: C, 62.70; H, 5.51; N, 5.62. Found: C, 62.85; H, 5.61; N, 5.42.

*4-(4-(2-(4-((Benzo[d][1,3]dioxol-5-yloxy)methyl)phenyl)ethyl)piperazin-1-yl)-3-fluorobenzonitrile* (**18**)*.* Yield: 75%, m.p. 182–183 °C (HCl salt); ^1^H-NMR (400 MHz, DMSO-*d*_6_) δ in ppm: 11.54 (s, 1H, N^+^H), 7.77 (dd, *J* = 13.1, 1.2 Hz, 1H, Ar-H), 7.63 (dd, *J* = 8.4, 1.2 Hz, 1H, Ar-H), 7.40 (d, *J* = 8.0 Hz, 2H, Ar-H), 7.30 (d, *J* = 8.0 Hz, 2H, Ar-H), 7.25 (t, *J* = 8.4 Hz, 1H, Ar-H), 6.80 (d, *J* = 8.5 Hz, 1H, Ar-H), 6.68 (d, *J* = 2.5 Hz, 1H, Ar-H), 6.43 (dd, *J* = 8.5, 2.5 Hz, 1H, Ar-H), 5.95 (s, 2H, CH_2_), 5.00 (s, 2H, CH_2_), 3.72 (d, *J* = 12.0 Hz, 2H, CH_2_), 3.64 (d, *J* = 12.0 Hz, 2H, CH_2_), 3.45–3.09 (m, 8H, CH_2_); ^13^C-NMR (101 MHz, DMSO-*d*_6_) δ in ppm: 154.39, 153.64, 151.94, 147.83, 142.35, 141.17, 136.53, 135.68, 129.90, 128.65, 127.98, 120.02, 119.92, 119.77, 118.07, 107.95, 106.17, 103.59, 100.94, 98.06, 69.69, 56.02, 50.43, 46.10, 28.88; Anal. Calc. for C_27_H_26_FN_3_O_3_·1HCl: C, 65.38; H, 5.49; N, 8.47. Found: C, 65.12; H, 5.45; N, 8.24.

*1-(2-(4-((Benzo[d][1,3]dioxol-5-yloxy)methyl)phenyl)ethyl)-4-(2-chlorophenyl)piperazine* (**19**)*.* Yield: 78%, m.p. 176–177 °C (HCl salt); ^1^H-NMR (400 MHz, DMSO-*d*_6_) δ in ppm: 11.27 (s, 1H, N^+^H), 7.46 (dd, *J* = 8.0, 1.6 Hz, 1H, Ar-H), 7.41 (d, *J* = 8.0 Hz, 2H, Ar-H), 7.35 (td, *J* = 8.0, 1.6 Hz, 1H, Ar-H), 7.31 (d, *J* = 8.0 Hz, 2H, Ar-H), 7.23 (dd, *J* = 8.0, 1.2 Hz, 1H, Ar-H), 7.12 (td, *J* = 8.0, 1.2 Hz, 1H, Ar-H), 6.80 (d, *J* = 8.5 Hz, 1H, Ar-H), 6.69 (d, *J* = 2.5 Hz, 1H, Ar-H), 6.43 (dd, *J* = 8.5, 2.5 Hz, 1H, Ar-H), 5.95 (s, 2H, CH_2_), 5.01 (s, 2H, CH_2_), 3.66 (d, *J* = 8.4 Hz, 2H, CH_2_), 3.44 (d, *J* = 8.4 Hz, 2H, CH_2_), 3.41–3.08 (m, 8H, CH_2_); ^13^C-NMR (101 MHz, DMSO-*d*_6_) δ in ppm: 154.16, 148.35, 147.88, 141.70, 137.08, 136.20, 130.93, 129.20, 128.72, 128.50, 128.04, 125.29, 121.52, 108.47, 106.70, 101.46, 98.58, 70.22, 56.59, 51.66, 48.15, 29.51; Anal. Calc. for C_26_H_27_ClN_2_O_3_·1.25HCl: C, 62.89; H, 5.73; N, 5.64. Found: C, 62.95; H, 5.70; N, 5.47.

*1-(2-(4-((Benzo[d][1,3]dioxol-5-yloxy)methyl)phenyl)ethyl)-4-(4-chlorophenyl)piperazine* (**20**)*.* Yield: 70%, m.p. 164–165 °C (HCl salt); ^1^H-NMR (400 MHz, DMSO-*d*_6_) δ in ppm: 11.64 (s, 1H, N^+^H), 7.40 (d, *J* = 8.0 Hz, 2H, Ar-H), 7.33–7.26 (m, 4H, Ar-H), 7.03 (d, *J* = 9.0 Hz, 2H, Ar-H), 6.80 (d, *J* = 8.5 Hz, 1H, Ar-H), 6.69 (d, *J* = 2.5 Hz, 1H, Ar-H), 6.43 (dd, *J* = 8.5, 2.5 Hz, 1H, Ar-H), 5.95 (s, 2H, CH_2_), 5.00 (s, 2H, CH_2_), 3.83 (d, *J* = 11.4 Hz, 2H, CH_2_), 3.62 (d, *J* = 11.4 Hz, 2H, CH_2_), 3.40–3.09 (m, 8H, CH_2_); ^13^C-NMR (101 MHz, DMSO-*d*_6_) δ in ppm: 153.66, 148.37, 147.84, 141.19, 136.66, 135.65, 128.76, 128.66, 127.98, 123.52, 117.47, 107.96, 106.19, 100.95, 98.08, 69.72, 55.91, 50.33, 45.16, 28.89; Anal. Calc. for C_26_H_27_ClN_2_O_3_·2HCl: C, 59.61; H, 5.58; N, 5.35. Found: C, 59.66; H, 5.59; N, 5.19.

*1-(2-(4-((Benzo[d][1,3]dioxol-5-yloxy)methyl)phenyl)ethyl)-4-(3-chlorophenyl)piperazine* (**21**). Yield: 65%, m.p. 177–178 °C (HCl salt); ^1^H-NMR (400 MHz, DMSO-*d*_6_) δ in ppm: 11.60 (s, 1H, N^+^H), 7.40 (d, *J* = 8.0 Hz, 2H, Ar-H), 7.30 (d, *J* = 8.0 Hz, 2H, Ar-H), 7.25 (t, *J* = 8.4 Hz, 1H, Ar-H), 7.06 (t, *J* = 2.4 Hz, 1H, Ar-H), 6.97 (dd, *J* = 8.4, 2.4 Hz, 1H, Ar-H), 6.87 (dd, *J* = 7.6, 1.2 Hz, 1H, Ar-H), 6.80 (d, *J* = 8.5 Hz, 1H, Ar-H), 6.68 (d, *J* = 2.5 Hz, 1H, Ar-H), 6.43 (dd, *J* = 8.5, 2.5 Hz, 1H, Ar-H), 5.95 (s, 2H, CH_2_), 5.00 (s, 2H, CH_2_), 3.90 (d, *J* = 11.6 Hz, 2H, CH_2_), 3.60 (d, *J* = 11.6 Hz, 2H, CH_2_), 3.40–3.07 (m, 8H, CH_2_); ^13^C-NMR (101 MHz, DMSO-*d*_6_) δ in ppm: 154.16, 151.27, 148.35, 141.69, 137.16, 136.16, 134.42, 131.08, 129.17, 128.49, 119.66, 115.76, 114.64, 108.47, 106.70, 101.45, 98.58, 70.22, 56.42, 50.77, 45.31, 29.40; Anal. Calc. for C_26_H_27_ClN_2_O_3_·2HCl: C, 59.61; H, 5.58; N, 5.35. Found: C, 59.55; H, 5.58; N, 5.18.

*1-(2-(4-((Benzo[d][1,3]dioxol-5-yloxy)methyl)phenyl)ethyl)-4-(2,3-dichlorophenyl)piperazine* (**22**)*.* Yield: 60%, m.p. 177–178 °C (HCl salt). ^1^H-NMR (400 MHz, DMSO-*d*_6_) δ in ppm: 10.78 (s, 1H, N^+^H), 7.41–7.30 (m, 6H, Ar-H), 7.22 (dd, *J* = 7.2, 2.8 Hz, 1H, Ar-H), 6.79 (d, *J* = 8.5 Hz, 1H, Ar-H), 6.68 (d, *J* = 2.5 Hz, 1H, Ar-H), 6.43 (dd, *J* = 8.5, 2.5 Hz, 1H, Ar-H), 5.94 (s, 2H, CH_2_), 5.00 (s, 2H, CH_2_), 3.66 (d, *J* = 10.0 Hz, 2H, CH_2_), 3.27–3.04 (m, 10H, CH_2_); ^13^C-NMR (101 MHz, DMSO-*d*_6_) δ in ppm: 155.53, 151.34, 149.73, 143.08, 140.49, 137.62, 134.60, 130.60, 130.50, 129.89, 127.95, 127.19, 121.74, 109.86, 108.09, 102.83, 99.95, 71.58, 57.99, 53.07, 49.66, 30.95; Anal. Calc. for C_26_H_26_Cl_2_N_2_O_3_·1HCl: C, 59.84; H, 5.21; N, 5.37. Found: C, 60.04; H, 5.27; N, 5.16.

*1-(2-(4-((Benzo[d][1,3]dioxol-5-yloxy)methyl)phenyl)ethyl)-4-(5-chloro-2-methoxyphenyl)piperazine* (**23**). Yield: 65%, m.p. 177–178 °C (HCl salt); ^1^H-NMR (400 MHz, DMSO-*d*_6_) δ in ppm: 11.46 (s, 1H, N^+^H), 7.40 (d, *J* = 8.0 Hz, 2H, Ar-H), 7.30 (d, *J* = 8.0 Hz, 2H, Ar-H), 7.05 (dd, *J* = 8.7, 2.4 Hz, 1H, Ar-H), 6.99 (d, *J* = 8.7 Hz, 1H, Ar-H), 6.94 (d, *J* = 2.4 Hz, 1H, Ar-H), 6.80 (d, *J* = 8.5 Hz, 1H, Ar-H), 6.68 (d, *J* = 2.5 Hz, 1H, Ar-H), 6.43 (dd, *J* = 8.5, 2.5 Hz, 1H, Ar-H), 5.95 (s, 2H, CH_2_), 5.00 (s, 2H, CH_2_), 3.80 (s, 3H, OCH_3_), 3.60 (d, *J* = 10.8 Hz, 2H, CH_2_), 3.55 (d, *J* = 10.8 Hz, 2H, CH_2_), 3.39–3.08 (m, 8H, CH_2_); ^13^C-NMR (101 MHz, DMSO-*d*_6_) δ in ppm: 154.16, 151.12, 148.35, 141.69, 141.14, 137.14, 136.18, 129.18, 128.49, 124.99, 122.98, 118.68, 113.74, 108.47, 106.70, 101.45, 98.58, 70.22, 56.55, 56.27, 51.37, 47.01, 29.43; Anal. Calc. for C_27_H_29_ClN_2_O_4_·1.8HCl: C, 59.33; H, 5.68; N, 5.12. Found: C, 59.26; H, 5.79; N, 4.87.

*1-(2-(4-((Benzo[d][1,3]dioxol-5-yloxy)methyl)phenyl)ethyl)-4-(4-bromophenyl)piperazine* (**24**)*.* Yield: 72%, m.p. 173–174 °C (HCl salt); ^1^H-NMR (400 MHz, DMSO-*d*_6_) δ in ppm: 11.62 (s, 1H, N^+^H), 7.43–7.35 (m, 4H, Ar-H), 7.29 (d, *J* = 8.0 Hz, 2H, Ar-H), 6.97 (d, *J* = 9.0 Hz, 2H, Ar-H), 6.79 (d, *J* = 8.5 Hz, 1H, Ar-H), 6.67 (d, *J* = 2.5 Hz, 1H, Ar-H), 6.42 (dd, *J* = 8.5, 2.5 Hz, 1H, Ar-H), 5.94 (s, 2H, CH_2_), 4.99 (s, 2H, CH_2_), 3.82 (d, *J* = 11.3 Hz, 2H, CH_2_), 3.60 (d, *J* = 11.3 Hz, 2H, CH_2_), 3.38–3.07 (m, 8H, CH_2_); ^13^C-NMR (101 MHz, DMSO-*d*_6_) δ in ppm: 154.16, 149.23, 148.35, 141.69, 137.17, 136.16, 132.14, 129.17, 128.49, 118.40, 111.72, 108.47, 106.70, 101.45, 98.58, 70.22, 56.42, 50.79, 45.53, 29.40; Anal. Calc. for C_26_H_27_BrN_2_O_3_·2HCl: C, 54.95; H, 5.14; N, 4.93. Found: C, 54.93; H, 5.15; N, 4.76.

*1-(2-(4-((Benzo[d][1,3]dioxol-5-yloxy)methyl)phenyl)ethyl)-4-(2-(trifluoromethyl)phenyl)piperazine* (**25**)*.* Yield: 75%, m.p. 171–172 °C (HCl salt); ^1^H-NMR (400 MHz, DMSO-*d*_6_) δ in ppm: 11.35 (s, 1H, N^+^H), 7.60–7.56 (m, 6H, Ar-H), 7.31 (d, *J* = 8.0 Hz, 2H, Ar-H), 6.80 (d, *J* = 8.5 Hz, 1H, Ar-H), 6.69 (d, *J* = 2.5 Hz, 1H, Ar-H), 6.43 (dd, *J* = 8.5, 2.5 Hz, 1H, Ar-H), 5.95 (s, 2H, CH_2_), 5.01 (s, 2H, CH_2_), 3.63 (d, *J* = 11.2 Hz, 2H, CH_2_), 3.45–3.34 (m, 4H, CH_2_), 3.23–3.07 (m, 6H, CH_2_); ^13^C-NMR (101 MHz, DMSO-*d*_6_) δ in ppm: 154.17, 150.95, 148.35, 141.69, 137.11, 136.18, 134.37, 129.21, 128.49, 127.59, 127.54, 126.58, 124.91, 108.47, 106.70, 101.45, 98.58, 70.22, 56.49, 51.95, 50.22, 29.55; Anal. Calc. for C_27_H_27_F_3_N_2_O_3_·1HCl: C, 62.25; H, 5.42; N, 5.38. Found: C, 62.41; H, 5.40; N, 5.24.

*1-(2-(4-((Benzo[d][1,3]dioxol-5-yloxy)methyl)phenyl)ethyl)-4-(4-(trifluoromethyl)phenyl)piperazine* (**26**)*.* Yield: 55%, m.p. 176–177 °C (HCl salt); ^1^H-NMR (400 MHz, DMSO-*d*_6_) δ in ppm: 11.45 (s, 1H, N^+^H), 7.57 (d, *J* = 8.8 Hz, 2H, Ar-H), 7.40 (d, *J* = 8.0 Hz, 2H, Ar-H), 7.30 (d, *J* = 8.0 Hz, 2H, Ar-H), 7.17 (d, *J* = 8.8 Hz, 2H, Ar-H), 6.80 (d, *J* = 8.5 Hz, 1H, Ar-H), 6.69 (d, *J* = 2.5 Hz, 1H, Ar-H), 6.43 (dd, *J* = 8.5, 2.5 Hz, 1H, Ar-H), 5.95 (s, 2H, CH_2_), 5.00 (s, 2H, CH_2_), 4.03 (d, *J* = 11.6 Hz, 2H, CH_2_), 3.64 (d, *J* = 11.6 Hz, 2H, CH_2_), 3.43–3.06 (m, 8H, CH_2_); ^13^C-NMR (101 MHz, DMSO-*d*_6_) δ in ppm: 153.64, 151.95, 147.83, 141.18, 136.60, 135.66, 128.66, 127.98, 126.28, 114.88, 107.95, 106.18, 100.94, 98.06, 69.70, 55.94, 50.19, 44.21, 28.93; Anal. Calc. for C_27_H_27_F_3_N_2_O_3_·1.25HCl: C, 61.18; H, 5.37; N, 5.28. Found: C, 61.49; H, 5.42; N, 5.19.

*1-(4-(4-(2-(4-((Benzo[d][1,3]dioxol-5-yloxy)methyl)phenyl)ethyl)piperazin-1-yl)phenyl)ethanone* (**27**)*.* Yield: 60%, m.p. 183–184 °C (HCl salt); ^1^H-NMR (400 MHz, DMSO-*d*_6_) δ in ppm: 11.22 (s, 1H, N^+^H), 7.86 (d, *J* = 8.8 Hz, 2H, Ar-H), 7.40 (d, *J* = 8.0 Hz, 2H, Ar-H), 7.30 (d, *J* = 8.0 Hz, 2H, Ar-H), 7.08 (d, *J* = 8.8 Hz, 2H, Ar-H), 6.80 (d, *J* = 8.5 Hz, 1H, Ar-H), 6.68 (d, *J* = 2.5 Hz, 1H, Ar-H), 6.43 (dd, *J* = 8.5, 2.5 Hz, 1H, Ar-H), 5.95 (s, 2H, CH_2_), 5.00 (s, 2H, CH_2_), 4.09 (d, *J* = 11.6 Hz, 2H, CH_2_), 3.64 (d, *J* = 11.6 Hz, 2H, CH_2_), 3.36–3.10 (m, 8H, CH_2_), 2.48 (s, 3H, CH_3_); ^13^C-NMR (101 MHz, DMSO-*d*_6_) δ in ppm: 195.72, 153.63, 152.54, 147.82, 141.17, 136.52, 135.67, 130.00, 128.65, 127.97, 127.78, 113.87, 107.94, 106.17, 100.93, 98.05, 69.68, 55.94, 50.25, 43.92, 28.94, 26.13; Anal. Calc. for C_28_H_30_N_2_O_4_·1.5HCl: C, 65.52; H, 6.19; N, 5.46. Found: C, 65.78; H, 6.47; N, 5.31.

*1-(2-(4-((Benzo[d][1,3]dioxol-5-yloxy)methyl)phenyl)ethyl)-4-phenylpiperidine* (**28**)*.* Yield: 75%, m.p. 183–184 °C (HCl salt); ^1^H-NMR (400 MHz, DMSO-*d*_6_) δ in ppm: 11.04 (s, 1H, N^+^H), 7.59–7.08 (m, 9H, Ar-H), 6.80 (d, *J* = 8.5 Hz, 1H, Ar-H), 6.69 (d, *J* = 2.5 Hz, 1H, Ar-H), 6.44 (dd, *J* = 8.5, 2.5 Hz, 1H, Ar-H), 5.95 (s, 2H, CH_2_), 5.01 (s, 2H, CH_2_), 3.64 (d, *J* = 11.6 Hz, 2H, CH_2_), 3.30–3.00 (m, 6H, CH_2_), 2.90–2.78 (m, 1H, CH), 2.10–2.08 (m, 4H, CH_2_); ^13^C-NMR (101 MHz, DMSO-*d*_6_) δ in ppm: 153.85, 148.04, 144.41, 141.38, 136.98, 135.83, 128.86, 128.70, 128.18, 126.71, 108.16, 106.39, 101.14, 98.27, 69.91, 56.73, 52.08, 29.93, 29.26; Anal. Calc. for C_27_H_29_NO_3_·1HCl: C, 71.75; H, 6.69; N, 3.10. Found: C, 71.25; H, 6.69; N, 2.98.

*1-(2-(4-((Benzo[d][1,3]dioxol-5-yloxy)methyl)phenyl)ethyl)-4-phenylpiperidin-4-ol* (**29**)*.* Yield: 80%, m.p. 194–195 °C (HCl salt); ^1^H-NMR (400 MHz, DMSO-*d*_6_) δ in ppm: 11.11 (s, 1H, N^+^H), 7.55–7.20 (m, 9H, Ar-H), 6.80 (d, *J* = 8.5 Hz, 1H, Ar-H), 6.69 (d, *J* = 2.5 Hz, 1H, Ar-H), 6.43 (dd, *J* = 8.5, 2.5 Hz, 1H, Ar-H), 5.95 (s, 2H, CH_2_), 5.47 (s, 1H, OH), 5.00 (s, 2H, CH_2_), 3.49 (d, *J* = 11.0 Hz, 2H, CH_2_), 3.43–3.06 (m, 8H, CH_2_), 1.82 (d, *J* = 11.0 Hz, 2H, CH_2_); ^13^C-NMR (101 MHz, DMSO-*d*_6_) δ in ppm: 153.65, 147.88, 147.83, 141.17, 136.77, 135.61, 128.67, 128.03, 127.95, 126.77, 124.51, 107.95, 106.18, 100.93, 98.07, 69.71, 68.00, 56.32, 48.25, 34.96, 29.17; Anal. Calc. for C_27_H_29_NO_4_·1HCl: C, 69.29; H, 6.46; N, 2.99. Found: C, 69.20; H, 6.45; N, 2.85.

### 3.2. In Vitro Cytotoxic Assay

#### 3.2.1. Cell Culture

PC-3 and RWPE-1 cells were cultured in Dulbecco’s modification Eagle’s medium (DMEM, Invitrogen, Carlsbad, CA, USA) supplemented with 10% fetal bovine serum (FBS, Hyclone, Logan, UT, USA), 100 U/mL penicillin and 0.1 mg/mL streptomycin (Invitrogen). DU145 cells were cultured in RPMI1640 media supplemented with 10% fetal bovine serum (FBS, Hyclone), 100 U/mL penicillin and 0.1 mg/mL streptomycin (Invitrogen). LNCaP cells were cultured in F12 media supplemented with 10% fetal bovine serum (FBS, Hyclone), 100 U/mL penicillin and 0.1 mg/mL streptomycin (Invitrogen). The cells were incubated at 37 °C in a humidified atmosphere with 5% CO_2_.

#### 3.2.2. Assessment of Antitumor Activity by CCK-8 Assay

Cell proliferation was measured with the Cell Counting Kit-8 (CCK-8) assay kit (Dojindo Corp., Kumamoto, Japan). Cells were harvested during logarithmic growth phase and seeded in 96-well plates at a density of 1 × 10^5^ cells/mL, and cultured at 37 °C in a humidified incubator (5% CO_2_) for 24 h, followed by exposure to various concentrations of compounds tested for 24 h. Subsequently 10 μL of CCK-8 (Dojindo) was added to each well, the cells were then incubated for an additional 1 h at 37 °C to convert WST-8 into formazan. Cell growth inhibition was determined by measuring the absorbance (Abs) at λ = 450 nm using amicroplate reader. Three independent experiments were performed. Cell growth inhibition was calculated according to the following equation:

Growth inhibition = (1 − OD of treated cells/OD of control cells) × 100%

The half maximal inhibitory concentrations (IC_50_) were obtained from linear regression analysis of the concentration-response curves plotted for each tested compound.

## 4. Conclusions

In summary, this study reported the synthesis and biological evaluation against three human prostate cancer cells and human prostate epithelial cells of a novel class of arylpiperazine derivatives. The results showed that the majority of the compounds exhibited excellent selective activity for LNCaP cells over the other tested cancer cells. The compounds with methyl (**9**) or fluoro (**15**) groups at the *o*-position on the phenyl group demonstrated a relatively strong cytotoxicity against LNCaP cells. It would be of interest to develop arylpiperazine derivatives for the treatment of the corresponding tumors. Designing more efficient derivatives from arylpiperazines based on the current study may successfully lead to the development of a potent anti-cancer agent. Further research involving other class of arylpiperazine derivatives, and preparation of analogs with aryl groups instead of a 1,3-benzodioxolyl group are in progress.
